# Abnormal resting state effective connectivity within the default mode network in major depressive disorder: A spectral dynamic causal modeling study

**DOI:** 10.1002/brb3.732

**Published:** 2017-06-04

**Authors:** Liang Li, Baojuan Li, Yuanhan Bai, Wenlei Liu, Huaning Wang, Hoi‐Chung Leung, Ping Tian, Linchuan Zhang, Fan Guo, Long‐Biao Cui, Hong Yin, Hongbing Lu, Qingrong Tan

**Affiliations:** ^1^ School of Biomedical Engineering Fourth Military Medical University Xi'an Shaanxi China; ^2^ Department of Psychiatry Xijing Hospital Fourth Military Medical University Xi'an Shaanxi China; ^3^ Department of Radiology Xijing Hospital Fourth Military Medical University Xi'an Shaanxi China; ^4^ Department of Psychology Stony Brook University Stony Brook NY USA

**Keywords:** default mode network, effective connectivity, major depressive disorder, resting state functional magnetic resonance imaging, spectral dynamic causal modeling

## Abstract

**Introduction:**

Understanding the neural basis underlying major depressive disorder (MDD) is essential for the diagnosis and treatment of this mental disorder. Aberrant activation and functional connectivity of the default mode network (DMN) have been consistently found in patients with MDD. It is not known whether effective connectivity within the DMN is altered in MDD.

**Objects:**

The primary object of this study is to investigate the effective connectivity within the DMN during resting state in MDD patients before and after eight weeks of antidepressant treatment.

**Methods:**

We defined four regions of the DMN (medial frontal cortex, posterior cingulate cortex, left parietal cortex, and right parietal cortex) for each participant using a group independent component analysis. The coupling parameters reflecting the causal interactions among the DMN regions were estimated using spectral dynamic causal modeling (DCM).

**Results:**

Twenty‐seven MDD patients and 27 healthy controls were included in the statistical analysis. Our results showed declined influences from the left parietal cortex to other DMN regions in the pre‐treatment patients as compared with healthy controls. After eight weeks of treatment, the influence from the right parietal cortex to the posterior cingulate cortex significantly decreased.

**Conclusion:**

These findings suggest that the reduced excitatory causal influence of the left parietal cortex is the key alteration of the DMN in patients with MDD, and the disrupted causal influences that parietal cortex exerts on the posterior cingulate cortex is responsive to antidepressant treatment.

## 1 INTRODUCTION

Major Depressive Disorder (MDD) is a psychiatric disorder characterized by persistent symptoms that interfere with daily life (American Psychiatric Association, 2000) and it is an increasing burden to society. However, effective diagnosis, treatment and prevention of the disorder have remained elusive. The main challenge appears to be our limited understanding of the primary underlying mechanisms of depression. Recently, there has been a growing optimism that functional neuroimaging may help us answer key questions about the pathophysiology of this disorder.

Previous neuroimaging studies have highlighted the involvement of default mode network (DMN) in the pathophysiology of MDD (Whitfield‐Gabrieli & Ford, 2012). The DMN consists of a specific set of regions including the midline cortical regions within the posterior cingulate cortex, precuneus, medial prefrontal cortex and lateral parietal regions (Raichle et al., 2001). These regions exhibit high metabolic activity at rest and during passive sensory processing tasks, while being deactivated during the performance of goal‐directed cognitive tasks (Buckner, Andrewshanna, & Schacter, 2008; Greicius, Krasnow, Reiss, & Menon, 2003). The DMN has been associated with self‐referential processes (Broyd et al., 2009; Gusnard, Akbudak, Shulman, & Raichle, 2001) and may be separable into anterior (ventromedial prefrontal cortex) and posterior (posterior cingulate cortex) components (Andrewshanna, Smallwood, & Spreng, 2014; Uddin, Kelly, Biswal, Castellanos, & Milham, 2009). It has also been implicated among the most discriminating networks classifying MDD patients from healthy controls (Zeng et al., 2012). Previous analyses of positron emission tomography and functional magnetic resonance imaging (fMRI) have revealed dysfunction of the anterior DMN regions in MDD patients, such as the increased metabolic activity in subgenual prefrontal cortex(Li et al., 2013; Manoliu et al., 2013; Mayberg, 1997, 2003), and the increased functional connectivity of DMN in subgenual anterior cingulate cortex and thalamus (Greicius et al., 2007). In the posterior regions of DMN, the functional connectivity decreased compared with healthy controls (Guo et al., 2014; Zhu et al., 2012). In addition, the functional connection between posterior cingulate cortex and bilateral caudate is reduced(Bluhm et al., 2009).

Efforts have also been made to investigate the effects of antidepressants on brain activity and connectivity, which may provide a potential therapeutic targets for MDD. Restored functional connectivity of the DMN has been observed in patients following antidepressant treatment (Delaveau et al., 2011; Fang et al., 2015; Wang et al., 2015). Although aberrant functional connectivity of the posterior DMN region was normalized with the remission of symptoms, the heightened functional connectivity of the anterior DMN region persisted in remitted MDD patients(Li et al., 2013).

Traditional functional connectivity measures correlations between brain regions based upon time series, without providing directed or causal interactions underlying the observed correlations (Friston, Harrison, & Penny, 2003). Thus, it remains unknown whether causal interactions within the DMN are also impaired in patients with MDD. In addition, it is unclear whether abnormalities in causal interactions could be restored after antidepressant treatment. Effective connectivity analysis on fMRI time series, such as structural equation modeling (Bavelier et al., 2000), Granger causality analysis (Goebel, Roebroeck, Kim, & Formisano, 2003) and dynamic causal modeling (Friston et al., 2003), offers a mechanistic description of causal interactions between different brain regions (Friston, 2011). Among all the effective connectivity methods, dynamic causal modeling (DCM) performs better than the others on modeling the neuronal coupling of fMRI data (Friston, 2009). Without any driving input, the DCM model for resting state fMRI can be estimated using stochastic DCM (Li et al., 2011) and spectral DCM (Friston, Kahan, Biswal, & Razi, 2014). As an extension of traditional deterministic DCM, stochastic DCM could estimate hidden neuronal fluctuations and model effective connectivity among brain regions at rest (Li et al., 2011). More importantly, stochastic DCM can be used to model effective connectivity among brain regions at rest. However, it suffers from unstable model inversion and high computation cost caused by evaluating neuronal variations in the time domain. The spectral DCM, instead, estimates effective connectivity based on correlation functions in the frequency domain, and therefore benefits from stable estimation and high computational efficiency. These features make spectral DCM a powerful tool for comparing directionality and couplings within an endogenous network between different groups of subjects (e.g. patients and controls).

In the present study, we hypothesized that effective connections between the DMN regions would be altered in patients with MDD, and part of the effective connections could be recovered after antidepressant treatment. By using the spectral DCM, we investigated DMN effective connectivity of pre‐treatment MDD patients, the same patients after eight weeks of antidepressant treatment and matched healthy controls. The DMN regions for each subject were first identified by spatial independent component analysis (McKeown & Sejnowski, 1998). Then the coupling parameters reflecting the causal (directed) interactions of the DMN regions were estimated using spectral DCM. Finally, the coupling parameters between the DMN regions were compared at the group level, and the relationship between coupling parameters and the clinical scores were analyzed.

## 2 MATERIALS AND METHODS

### 2.1 Participants

Thirty‐five patients with MDD and 31 healthy controls with no history of neurological or psychiatric disease were screened for this study. MDD was diagnosed by psychiatrists based on the DSM‐IV criteria, including the Structured Clinical Interview. All patients were antidepressant drug‐free for at least three months before participating this study. All subjects completed the 17‐item version of the Hamilton Rating Scale for Depression (HAMD) and the Hamilton Rating Scale for Anxiety (HAMA). Patients with an HAMD score <18 or with other concurrent psychiatric were excluded from the current study. Finally, 27 MDD patients and 27 healthy controls were included in the analyses.

Baseline resting‐state fMRI images were first collected from the unmedicated MDD subjects and the controls. The patients were then treated with venlafaxine hydrochloride capsules (150–225 mg/day) for eight weeks (60 ± 10 days). Seven patients (six female, one male, aged 36.43 ± 11.24) had a combined treatment of venlafaxine with repetitive transcranial magnetic stimulation (rTMS) on the left dorsolateral prefrontal cortex. The combined treatment has shown an antidepressant effect in the previous stduy(Padberg & George, 2009). The rTMS treatment lasted for 14 days. The pulses of the rTMS were delivered with a frequency of 10 Hz and at 110% of the resting motor threshold (measured once, before the treatment series). Stimulation was delivered each day in 50 trains of 4 s each, and 30 s inter stimulus intervals.

After eight weeks of antidepressant treatment, the patients had a second fMRI scan and repeated the structured clinical interview for DSM‐IV. Table 1 shows the final demographic and clinical characteristics of participants: the control subjects, the pre‐treatment MDD group and post‐treatment MDD group. The post‐treatment group contains the same patients as the pre‐treatment group after eight weeks of antidepressant drug alone or combined with rTMS treatment. This study was approved by the ethics committee of Fourth Military Medical University. Written informed consent was obtained from each subject before participation.

**Table 1:** Demographic and clinical characteristics of the participants

Characteristics	Pre‐treatment	Post‐treatment	Healthy control	*p* value^a^
Gender (M/F)	8/19	8/19	8/19	–
Age (Years)	35.63 ± 12.67	35.63 ± 12.67	33.30 ± 12.47	0.4982
Education (Years)	10.70 ± 3.86	10.70 ± 3.86	16.19 ± 4.58	<.001
HAMD	22.15 ± 3.85	7.30 ± 3.76	2.41 ± 3.39	<.001
HAMA	20.75 ± 3.89	6.30 ± 3.20	2.04 ± 3.04	<.001

a HAMD and HAMA were tested with a one‐way ANOVA.

### 2.2 Data acquisition

Resting‐state fMRI data were collected using a GE Discovery MR750 3.0 Tesla whole‐body scanner (GE Medical Systems, Milwaukee, WI, USA) in Xijing hospital. Subjects were instructed to lie still inside the scanner, close their eyes, stay awake and try not to think about anything special. For each subject, 210 volumes of fMRI images were acquired with an echo‐planar imaging (EPI) sequence using the following parameters: repetition time = 2000 ms, echo time = 30 ms, field of view = 240 mm × 240 mm, matrix = 64 × 64, flip angle = 90°, number of slices = 45, slice thickness = 3.5 mm, spacing = 0.0 mm.

### 2.3 Preprocessing

Resting‐state fMRI data were preprocessed using the statistical parametric mapping software package (SPM8: RRID:SCR_007037; version: 6313; http://www.fil.ion.ucl.ac.uk/spm/software/spm8). For each dataset, the first five functional volumes were discarded to allow the fMRI signal stabilized. The remaining 205 volumes of each dataset went through the preprocessing steps including slice timing correction, realignment to compensate for motion, and affine‐only normalization to the standard EPI template in the Montreal Neurological Institute (MNI, Quebec, Canada) space (2 mm isotropic voxels). Finally, the resulting data were spatially smoothed with a Gaussian Kernel filter of 8 mm full‐width half‐maximum kernel.

### 2.4 Group independent component analysis

After data preprocessing, group independent component analysis (ICA) was performed to decompose the fMRI images into spatially independent networks using the Group ICA Toolbox (GIFT: RRID:SCR_001953; version: 2.0a; http://icatb.sourceforge.net/). The number of independent components was first estimated from the fMRI data of all subjects with the minimum description length (MDL) criterion. The fMRI images were then decomposed into the estimated number of spatially independent components. Finally, all the independent component maps were spatial sorted according to the default mode template rDMN_ICA_REST_3x3x3.nii (Franco, Pritchard, Calhoun, & Mayer, 2009) provided by the GIFT software. The independent component that best fit this template was identified as representing the spatial pattern of the DMN. In addition, the functional connectivity within the DMN was analysed by comparing the DMN components between the patients with MDD and the healthy controls.

### 2.5 Selection of regions of interest

For each subject, four regions of interest (ROIs) including the medial frontal cortex (MFC), posterior cingulate cortex (PCC), left parietal cortex (LPC), and right parietal cortex (RPC) were selected according to the spatial pattern of the subject‐specific independent component representing the DMN. ROIs were defined as spheres with a radius of 8 mm centered at the peak coordinate from the identified DMN component map of each subject using xjView: RRID:SCR_008642 (http://www.alivelearn.net/xjview). Table S1 provided detail information on the locations of the ROIs for each subject. This procedure ensured that DCM analysis was performed on those regions identified as a functionally connected network for each subject.

Time series from the ROIs were created as the residuals of a general linear model (GLM). This model included the following regressors: (1) the six rigid body realignment parameters to model the movement correlated effects; (2) one constant regressor to model the baseline that was used in the extractions to constrain the extraction of BOLD fMRI time series within the brain; and (3) cosine basis functions to model possible signal drift and aliased respiratory and cardiac signals; and (4) a high‐pass filter of 1/128 Hz to remove possible ultraslow fluctuations.

### 2.6 Spectral dynamic causal modeling

The spectral DCM analysis was performed using DCM12 routine implemented in SPM12. After extracting the resting‐state fMRI time series from all four ROIs, a fully‐connected model which has bi‐directional connections between any pair of ROIs was specified for each subject. We were modeling on the resting‐state fMRI data. No exogenous input was included in the model. Thus the specified model has 2^4^ = 16 free parameters describing the effective connections among the four ROIs and the self‐connections within the ROIs. Parameters of the fully‐connected model were then estimated. Unlike traditional DCM analysis, the hidden neuronal states in the time domain were no longer estimated in spectral DCM. Instead, the convolution kernel representation of DCM was transformed to a spectral representation in the frequency domain, which made the inversion of DCM more computationally efficient than stochastic DCM. Thus all coupling parameters of DMN models were fitted with an estimation procedure using second‐order statistics characterizing spectral densities over frequencies. For more detail, please refer to the technical note from Friston et al. (Friston et al., 2014). Finally, Bayesian model selection (BMS) was performed using a post‐hoc optimization method to select the ‘winning’ model with the best balance between accuracy and complexity (Stephan, Penny, Daunizeau, Moran, & Friston, 2009). Through the BMS the optimal model gains maximal generalizability (Pitt, Myung, & Zhang, 2002).

We assumed that the three groups did not share the same model structure. Therefore, the BMS was done on each group separately. The BMS was performed with the spm_dcm_post_hoc function. When free parameters were equal or more than 16, this routine would implement a greedy search, which entailed searching over all permutations of the eight parameters whose removal produced the smallest reduction in model evidence. This procedure was repeated until all eight parameters were retained in the optimal model or there were no more parameters to consider (Friston & Penny, 2011). The specified fully‐connected model contained 2^4^ = 16 free parameters; thus, the BMS resulted in a 2^8^ = 256 reduced model space. Then the reduced model with the greatest model evidence was selected as the optimal model, and the corresponding parameters for the optimal model were also estimated.

### 2.7 Statistical analyses

The parameters for the optimal model were tested by one‐sample *t*‐test to identify significant effective connectivity in each group. Correction for false discovery rate (FDR) was applied to the results of one‐sample *t*‐test by the function (p.adjust) in R: RRID:SCR_001905 (www.R-project.org). In order to investigate abnormalities in effective connectivity within the DMN, the coupling parameters of different groups were analyzed using factorial ANOVA (using function lm and anova in R) with age, education and group as factors. Finally, the effects of different treatment methods on the coupling parameters within the DMN were examined by comparing the coupling parameters of the pre‐treatment MDD group and the post‐treatment MDD group with paired *t*‐test. The treatment methods were added as a factor in ANOVA to test whether the changes of coupling parameters could be explained by treatment method. Based on the coupling parameters in both pre‐treatment and post‐treatment patients, we performed the linear regression and calculated the coefficient of determination to test if the coupling parameters were able to linearly explain the clinical socres (HAMA and HAMD).

## 3 RESULTS

### 3.1 Group independent component analysis

**Figure 1:**
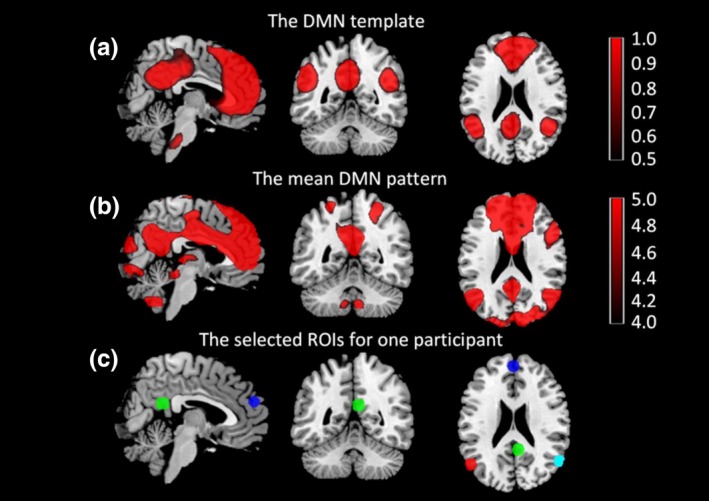
DMN template, mean DMN pattern and selected ROIs. (a) DMN template (rDMN_ICA_REST_3x3x3.nii), (b) mean DMN independent component for all subjects, (c) Selected DMN regions used for DCM analysis. The blue sphere represents the region of MFC, the green sphere illustrates the region of PCC, the red sphere represents the region of LPC, and the cyan sphere shows the region of RPC

The estimated number of independent components was 39 according to the MDL criterion. The resulting components were sorted by their spatial correlation with the DMN template. The mean independent component that best fit the DMN template with a regression coefficient of 0.27144 was identified as the default mode component. Figure 1a shows the spatial pattern of the DMN template. The mean DMN component identified for all the subjects is shown in Figure 1b. Figure 1a,b show clearly that both the DMN template and the DMN component mainly contains four regions of the DMN including the MFC, PCC, LPC, and RPC. Subject‐specific ROIs were defined as 8 mm spheres centered at the peak value of each of the four regions for each subject. Figure 1c shows the locations of the four sphere ROIs of one subject. In addition, the coordinates of selected ROIs of all participants are provided in Table S1. In order to make sure that the locations of the subject‐specific ROIs do not differ between different groups, the Euclidean distance between the individual ROI and the average ROI for all the subjects were calculated. The mean Euclidean distances of different groups were then compared using a Kruskal‐Wallis test ran in prism 6. There were no significant differences in the Euclidean distance of the pre‐treatment MDD, post‐treatment MDD, and the control groups. Details are listed in the Table S2.

**Figure 2:**
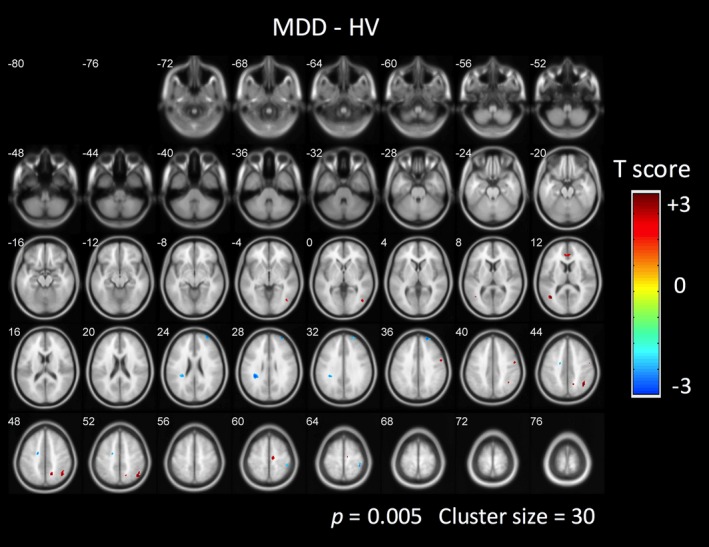
The difference of the functional connectivity within the DMN. The functional connectivity in the red region is significantly higher in the MDD patients than the healthy controls. The functional connectivity in the blue region is significantly lower in the MDD patients than the healthy controls

In addition, the functional connectivity of the DMN was compared by performing unpaired t‐test on the DMN components between pre‐treatment patients and healthy volunteers. Figure 2 shows the results of unpaired t‐test. The functional connectivity of two regions within the DMN were significantly different between the two groups (*p* = .005, uncorrected). The functional connectivity of the patients with MDD was significatly increased in the BA 24 (MNI coordinate: x = −2, y = 38, z = 10). The functional connectivity in the patients with MDD was significatly increased in the right inferior parital cortex (MNI coordinate: x = 40, y = −58, z = 46). But these results did not survive FDR correction.

### 3.2 Bayesian model selection

**Figure 3:**
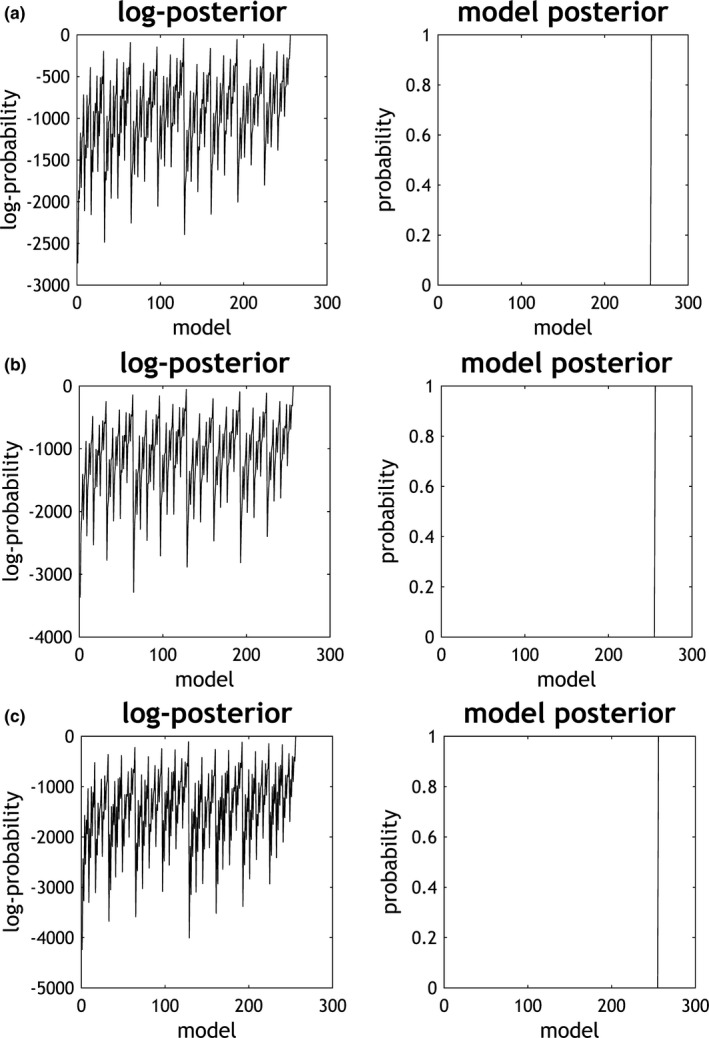
Results of Bayesian model selection for three groups. (a) Pre‐treatment patients, (b) post‐ treatment patients, and (c) healthy controls. Left: the log‐posterior of all reduced models; right: the posterior probabilities of all evaluated models. The fully connected model is the best model with a posterior probability of almost one

The BMS in SPM12 implemented a greedy search in the 256 reduced model spaces to find the optimal model for each group. Figure 3a–c give the results of this BMS for patients pre‐treatment MDD patients, post‐treatment MDD patients and healthy controls. The left column of Figure 3a shows the distribution of log Bayesian model evidence over the 256 reduced models for pre‐treatment patients. The fully connected model has the highest evidence. The right column of Figure 3a gives the posterior probability distribution of all reduced models. The fully connected model was recognized as the optimal model with a probability of almost one. Similarly, Figure 3b,c indicate that the fully connected model is the optimal model for pre‐treatment and the control group, suggesting that all three groups of subjects share the same fully connected model structure.

### 3.3 Statistical analysis on effective connectivity

**Figure 4:**
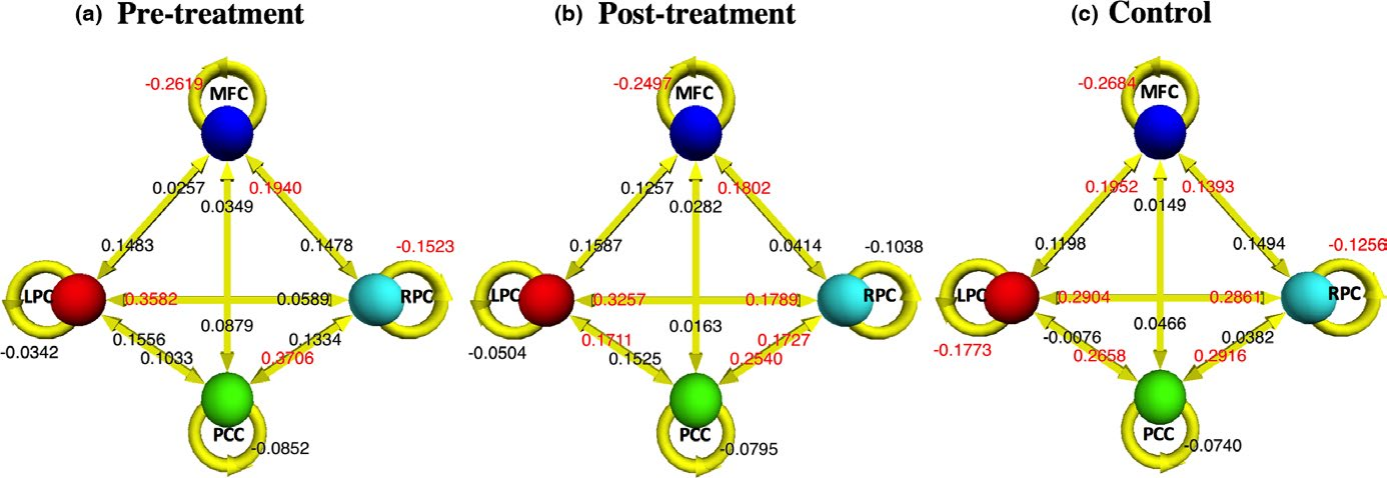
Mean coupling parameters (in Hz) within the DMN for three groups, i.e., (a) pre‐treatment patients, (b) post‐treatment patients, and (c) healthy controls. The coupling parameters labeled in red are significantly different from zero (*p* < .05, FDR corrected)

After identifying the fully connected model as the optimal model, the endogenous connections (DCM.Ep.A) within the DMN of different groups were compared. We first performed a one‐sample *t*‐test to determine which coupling parameters were significantly different from zero in each group. The FDR correction was performed on the one‐sample *t*‐test results. Figure 4 displays significant connections (*p *<* *.05, FDR corrected) for the pre‐treatment patients, post‐treatment patients, and healthy controls, respectively.

**Figure 5:**
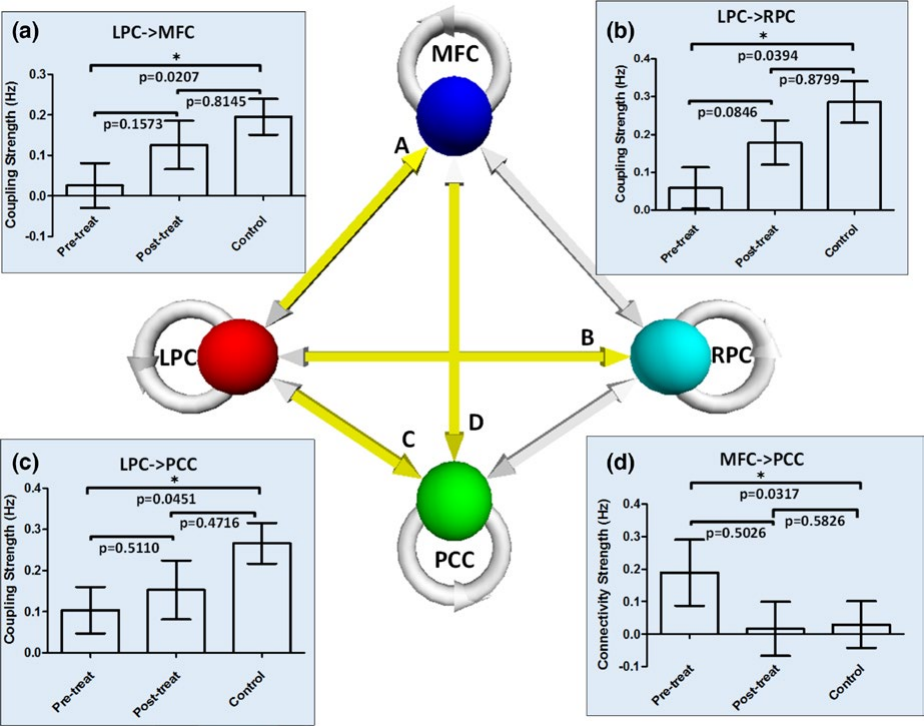
The Significant difference in the strength of coupling parameter between pre‐treatment/post‐treatment group and control group with factorial ANOVA. The coupling parameters with significant differences (p<.05, uncorrected) between pre‐treatment and control group or between post‐treatment and control group were marked in the figure. (a) Coupling parameter from LPC to MFC, (b) Coupling parameter from LPC to RPC, (c) Coupling parameter from LPC to PCC and (d) Coupling parameter from MFC to PCC. The bars show the mean and SEM of the coupling parameter

Considering age and education as factors, significant differences between the pre‐treatment MDD patients and controls in coupling parameters were identified using analysis of variance (ANOVA). The same analysis was also performed between the post‐treatment MDD patients and controls. As is shown in Figure 5a–c, the coupling parameters from LPC to MFC, PCC and RPC in the pre‐treatment MDD group were significantly lower than those of the control group (*p *<* *.05, uncorrected). However, the differences between the post‐treatment group and the control group were not significant. As shown in Figure 5d, the coupling parameter from MFC to PCC in the pre‐treatment group was significantly higher (*p *<* *.05) than that of the control group. The mean connections from LPC to MFC, PCC and RPC increased in the post‐treatment group, but these increased connections do not show the significant difference between the pre‐treatment MDD and post‐treatment MDD group with paired *t*‐test.

**Figure 6:**
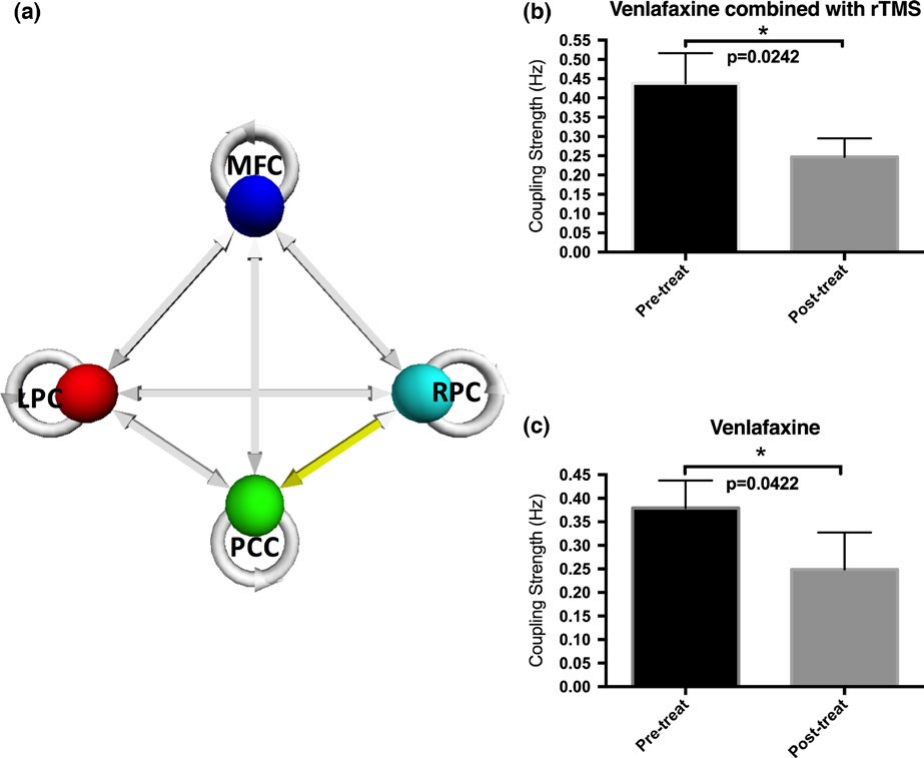
The significant changed (p<.05, uncorrected) coupling parameter from RPC to PCC between the pre‐treatment group and post‐treatment group. (a) The effective connection from RPC to PCC within DMN; (b) The coupling parameter changes for patients treated by the Venlafaxine combined with the rTMS; and (c) The coupling parameter changes for patients treated by the Venlafaxine alone

We performed paired *t*‐test on the coupling parameters between the pre‐treatment MDD group and the post‐treatment MDD group. The effective connection from RPC to PCC was significantly (*p *=* *.0406, uncorrected) reduced after eight weeks of antidepressant treatment. Then the pateints were seperated into Venlafaxine group and Venlafaxine combined with rTMS group according to the treatment methods. As shown in the Figure 6, the coupling parameters from RPC to PCC significantly reduced (*p *=* *.0422, uncorrected) in the Venlafaxine group. In the Venlafaxine combined with rTMS group, the coupling parameters from RPC to PCC also significantly reduced (*p *=* *.0242, uncorrected). The ANOVA test for changes of coupling parameters with treatment methods as a factor was not significant (*p *=* *.4269).

No significant linear correlations were found between the coupling parameters and clinical scores in the patients with MDD. The scatter plots of coupling parameters against HAMA and HAMD scores for pre‐treatment and post‐treatment group were shown in the Figures S1–S4.

## 4 DISCUSSION

In this study, we used spectral DCM to explore the causal interactions within the DMN in MDD patients and healthy controls during resting state. We identified how regions within the DMN interact with each other at resting state in pre‐treatment MDD patients, post‐treatment MDD patients, and healthy controls. Our analyses showed that the effective connections from the LPC to MFC, PCC and RPC were significantly lower in pre‐treatment MDD group than in the control group. In addition, the influence from the anterior section of the DMN to the posterior section was significantly greater in the MDD patients compared to the healthy controls. Finally, our results also revealed that the effective connection from the RPC to PCC was significantly decreased after eight weeks of antidepressant treatment.

### 4.1 Effective connectivity within the DMN in MDD patients

In the current study, we first used DCM to investigate whether causal interactions between regions within the DMN were changed in MDD patients. We found that the effective connection from MFC to PCC was enhanced in the pre‐treatment MDD group, while efferent causal influences from LPC to MFC, RPC and PCC were reduced in the patients. These findings further support the results of previous studies, in which abnormalities in functional connectivity of the DMN in MDD patients have been consistently reported.

Greicius et al. conducted one of the first studies to investigate resting‐state functional connectivity of the DMN in MDD patients. Using ICA, the authors found increased DMN connectivity in depressed subjects compared to healthy controls (Greicius et al., 2007). Gaffrey et al. investigated how a history of preschool depression may affect the developmental trajectory of the DMN. Using the PCC as a seed region, they examined the functional connectivity between the PCC and other brain regions. Children with a history of preschool depression exhibited increased PCC functional connectivity in the subgenual and anterior cingulate cortices (Gaffrey, Luby, Botteron, Repovs, & Barch, 2012). Elevated DMN functional connectivity in depressed patients was also detected when subjects were instructed to engage in externally‐focused thought (Belleau, Taubitz, & Larson, 2014). Additionally, adolescents with MDD exhibited persistent and elevated DMN connectivity, both at rest and during an emotion identification task (Ho et al., 2014).

In this study, we used DCM to further investigate whether causal interactions between regions within the DMN were also impaired in the MDD patients. Our findings suggest that not only undirected functional connectivity within the DMN was impaired in patients with MDD, but also how signals propagate from one region of the DMN to another was altered in these patients. In particular, the influences from the left part to other regions of this network were diminished while the interactions from the anterior part to the posterior part was enhanced.

### 4.2 The effects of antidepressant treatment on the effective connectivity within the DMN in MDD patients

The comparison between the pre‐ and post‐treatment MDD group shows a significant decrease in causal influence from RPC to PCC after eight weeks of antidepressant treatment, as shown in Figure 4. The elevated PCC connectivity in the DMN in MDD patients is reduced with the improvement of symptoms, suggesting this causal connection may be responsive to antidepressant treatment.

Our findings are in line with previous studies on patients following antidepressant treatment, in which decreased activation and restored functional connectivity of the DMN have been reported (Delaveau et al., 2011; Fang et al., 2015; Wang et al., 2015; Wu et al., 2011). Antidepressants also normalized the increased DMN functional connectivity in subjects with dysthymic disorder, a mild but long‐term form of depression (Posner et al., 2013).

We did not detect any significant increase in the connections from the LPC to MFC, RPC or PCC with the improvement of the symptoms, which were diminished in the pre‐treatment MDD group. This may due to the relatively small sample size in the current study. In fact, the results from one‐sample *t*‐test showed that the connection from LPC to RPC was significantly greater than zero in the post‐treatment MDD group, while this connection was not significantly greater than zero in the pre‐treatment MDD group, suggesting a trend towards increase after the antidepressant treatment. On the other hand, the reason that we did not observe any significant increase in the connections from LPC to other regions may be due to the effects of antidepressants observed in other studies (Lemogne et al., 2010). Specifically, abnormalities in the frontal cortex have been found to persist after treatment (Lemogne et al., 2010; Li et al., 2013; Wu et al., 2011). In our previous work, we investigated changes in DMN functional connectivity after 12 weeks of antidepressant treatment in MDD patients. Although depressed subjects exhibited increased functional connectivity in both anterior and posterior sub‐networks, aberrant connectivity of the posterior sub‐network was normalized with the remission of symptoms. In contrast, elevated functional connectivity of the anterior sub‐network persisted in remitted subjects. This indicates that antidepressants may not modulate the functional connectivity of the medial prefrontal cortex (Li et al., 2013).

### 4.3 Limitations

The present study has some limitations. The sample size of the MDD patients and matched control group was relatively small. Few patients received rTMS together with the antidepressant drug. Due to the sample size we did not explore the effects of the drug compared to drug plus rTMS treatment. More qualified subjects should be recruited in future studies to increase the reliability of the above observations and the effect of different treatment methods on the DMN. In addition, the education level was not matched between the control group and the patient group, and sex was not considered during recruitment. Finally, it would be best to collect fMRI data from healthy controls eight weeks after the first scan to confirm that they would have not demonstrated similar changes in brain connections as MDD patients did. We only scanned them once and assume that brain connectivity would not change significantly in a short period of time.

## 5 CONCLUSIONS

This is the first study that explores the effective connectivity within DMN using spectral dynamic causal modeling method in patients with MDD. Our work suggests that the impaired excitatory causal influence of LPC is the key alteration of the DMN in patients with MDD. The causal influence that the RPC exert on PCC is responsive to antidepressant treatment. Altered effective connectivity within DMN in patients with MDD may provide helpful information for the diagnosis and treatment of this disorder.

## CONFLICT OF INTERESTS

No conflict of interests.
