# Supplementary material for: Abnormal resting state effective connectivity within the default mode network in major depressive disorder: A spectral dynamic causal modeling study
Source: Brain Behav. 2017 Jun 4;7(7):e00732. doi: 10.1002/brb3.732 (PMC5516606; doi:10.1002/brb3.732)
Supplement: Supplementary file 1 [file BRB3-7-e00732-s001.docx]

**Supplementary Material**

Table 1 Coordinates in Montreal Neurological Institute (MNI) space obtained from independent component analysis at the single-subject level.

| **Subject** | **MFC** | | | **PCC** | | | **LPC** | | | **RPC** | | |
| --- | --- | --- | --- | --- | --- | --- | --- | --- | --- | --- | --- | --- |
|  | x | y | z | x | y | z | x | y | z | x | y | z |
| *Control* |  |  |  |  |  |  |  |  |  |  |  |  |
| C1 | 2 | 52 | 36 | 0 | -50 | 18 | -56 | -60 | 20 | 56 | -58 | 38 |
| C2 | 0 | 48 | 36 | 2 | -46 | 28 | -46 | -62 | 26 | 54 | -52 | 20 |
| C3 | 2 | 56 | 30 | 2 | -54 | 18 | -46 | -64 | 24 | 52 | -66 | 24 |
| C4 | -2 | 46 | 40 | -2 | -54 | 34 | -48 | -70 | 36 | 54 | -66 | 20 |
| C5 | 0 | 48 | 38 | 0 | -66 | 34 | -46 | -70 | 30 | 54 | -60 | 18 |
| C6 | -2 | 50 | 38 | 0 | -58 | 20 | -48 | -60 | 28 | 52 | -60 | 40 |
| C7 | -8 | 36 | 56 | 2 | -50 | 16 | -56 | -60 | 22 | 58 | -52 | 24 |
| C8 | 0 | 54 | 34 | -2 | -58 | 32 | -48 | -68 | 24 | 56 | -56 | 26 |
| C9 | 0 | 52 | 20 | 0 | -62 | 36 | -46 | -70 | 32 | 56 | -60 | 32 |
| C10 | -2 | 52 | 32 | 0 | -52 | 24 | -50 | -60 | 26 | 56 | -54 | 20 |
| C11 | -2 | 52 | 34 | 2 | -50 | 24 | -50 | -66 | 24 | 56 | -60 | 24 |
| C12 | -4 | 38 | 32 | 0 | -60 | 48 | -42 | -72 | 30 | 54 | -62 | 38 |
| C13 | 0 | 56 | 18 | -2 | -52 | 32 | -50 | -64 | 32 | 48 | -58 | 32 |
| C14 | 0 | 48 | 22 | 2 | -58 | 32 | -54 | -62 | 26 | 52 | -54 | 32 |
| C15 | 0 | 56 | 22 | 0 | -62 | 32 | -48 | -72 | 26 | 54 | -64 | 26 |
| C16 | -4 | 54 | 32 | 2 | -62 | 30 | -52 | -70 | 24 | 54 | -58 | 30 |
| C17 | 0 | 50 | 36 | 0 | -50 | 28 | -50 | -70 | 26 | 54 | -58 | 22 |
| C18 | 0 | 52 | 30 | 4 | -52 | 26 | -52 | -60 | 26 | 52 | -54 | 36 |
| C19 | -2 | 42 | 48 | -2 | -54 | 24 | -54 | -60 | 28 | 52 | -64 | 32 |
| C20 | 2 | 46 | 32 | 2 | -52 | 24 | -48 | -70 | 24 | 52 | -62 | 28 |
| C21 | 0 | 46 | 38 | 2 | -60 | 20 | -46 | -54 | 26 | 58 | -60 | 20 |
| C22 | 2 | 50 | 38 | 0 | -58 | 32 | -52 | -70 | 18 | 56 | -62 | 16 |
| C23 | 0 | 44 | 42 | -2 | -56 | 26 | -48 | -62 | 28 | 52 | -64 | 28 |
| C24 | 0 | 46 | 40 | 0 | -54 | 24 | -46 | -66 | 26 | 56 | -60 | 22 |
| C25 | 0 | 42 | 52 | 0 | -52 | 26 | -50 | -66 | 24 | 54 | -60 | 28 |
| C26 | -4 | 52 | 38 | 0 | -48 | 34 | -50 | -62 | 36 | 58 | -54 | 22 |
| C27 | 0 | 48 | 40 | 0 | -62 | 30 | -46 | -74 | 34 | 46 | -68 | 40 |
| *Before* |  |  |  |  |  |  |  |  |  |  |  |  |
| B1 | 4 | 40 | 46 | 0 | -52 | 34 | -44 | -72 | 26 | 46 | -56 | 30 |
| B2 | -2 | 52 | 18 | 0 | -54 | 28 | -48 | -68 | 36 | 56 | -58 | 32 |
| B3 | 0 | 48 | 40 | -2 | -44 | 26 | -52 | -64 | 28 | 54 | -58 | 38 |
| B4 | -2 | 48 | 36 | 2 | -48 | 26 | -54 | -58 | 22 | 52 | -66 | 26 |
| B5 | 0 | 48 | 40 | 2 | -60 | 24 | -48 | -69 | 30 | 48 | -66 | 38 |
| B6 | 0 | 52 | 34 | 2 | -56 | 22 | -48 | -70 | 31 | 60 | -56 | 24 |
| B7 | -2 | 40 | 46 | 0 | -54 | 28 | -46 | -68 | 40 | 54 | -62 | 34 |
| B8 | 2 | 50 | 42 | 2 | -50 | 24 | -46 | -58 | 32 | 52 | -66 | 26 |
| B9 | -4 | 50 | 32 | -2 | -52 | 30 | -48 | -68 | 28 | 52 | -66 | 28 |
| B10 | -2 | 54 | 24 | 2 | -44 | 30 | -52 | -66 | 32 | 54 | -60 | 30 |
| B11 | -2 | 50 | 32 | -4 | -48 | 32 | -48 | -70 | 28 | 52 | -64 | 26 |
| B12 | -2 | 50 | 28 | -4 | -52 | 30 | -50 | -62 | 38 | 60 | -48 | 30 |
| B13 | -2 | 48 | 44 | 2 | -56 | 28 | -44 | -66 | 48 | 50 | -56 | 28 |
| B14 | 0 | 46 | 40 | -2 | -50 | 28 | -42 | -66 | 38 | 54 | -52 | 40 |
| B15 | 0 | 54 | 34 | 2 | -60 | 26 | -44 | -66 | 36 | 56 | -64 | 32 |
| B16 | 0 | 48 | 30 | 0 | -50 | 22 | -52 | -58 | 34 | 52 | -60 | 38 |
| B17 | 2 | 50 | 30 | 0 | -52 | 26 | -48 | -64 | 28 | 52 | -66 | 30 |
| B18 | 2 | 52 | 28 | -2 | -56 | 32 | -56 | -56 | 34 | 54 | -62 | 26 |
| B19 | 0 | 44 | 40 | -4 | -52 | 30 | -46 | -62 | 40 | 56 | -58 | 32 |
| B20 | 2 | 48 | 42 | -2 | -52 | 24 | -46 | -70 | 36 | 52 | -60 | 34 |
| B21 | -2 | 44 | 36 | 2 | -54 | 28 | -54 | -62 | 18 | 54 | -62 | 30 |
| B22 | -4 | 38 | 54 | -4 | -54 | 26 | -44 | -60 | 25 | 58 | -58 | 24 |
| B23 | 2 | 50 | 38 | 0 | -54 | 28 | -50 | -66 | 32 | 56 | -60 | 30 |
| B24 | 2 | 52 | 32 | -2 | -52 | 24 | -52 | -66 | 28 | 54 | -58 | 26 |
| B25 | -4 | 48 | 40 | 2 | -50 | 22 | -46 | -68 | 34 | 40 | -68 | 40 |
| B26 | -2 | 50 | 38 | -4 | -60 | 22 | -46 | -64 | 42 | 50 | -60 | 26 |
| B27 | 0 | 52 | 32 | -4 | -56 | 28 | -50 | -62 | 32 | 54 | -60 | 34 |
| *After* |  |  |  |  |  |  |  |  |  |  |  |  |
| A1 | 2 | 54 | 24 | -4 | -52 | 34 | -46 | -70 | 34 | 52 | -64 | 28 |
| A2 | 0 | 54 | 20 | -2 | -50 | 28 | -50 | -60 | 28 | 52 | -66 | 26 |
| A3 | 0 | 46 | 44 | -6 | -56 | 38 | -50 | -64 | 28 | 48 | -72 | 30 |
| A4 | -4 | 50 | 36 | 4 | -48 | 26 | -52 | -62 | 24 | 50 | -66 | 32 |
| A5 | 0 | 48 | 40 | -2 | -56 | 24 | -44 | -68 | 28 | 48 | -66 | 30 |
| A6 | 0 | 36 | 56 | 2 | -64 | 26 | -52 | -70 | 26 | 60 | -54 | 20 |
| A7 | 0 | 52 | 40 | 2 | -54 | 20 | -52 | -58 | 36 | 56 | -60 | 34 |
| A8 | 0 | 44 | 42 | 2 | -58 | 26 | -46 | -72 | 26 | 52 | -64 | 26 |
| A9 | -4 | 52 | 36 | -4 | -50 | 30 | -48 | -68 | 32 | 50 | -64 | 34 |
| A10 | -2 | 48 | 42 | 4 | -56 | 16 | -50 | -64 | 34 | 48 | -52 | 26 |
| A11 | 2 | 50 | 32 | 0 | -48 | 30 | -50 | -66 | 26 | 54 | -58 | 24 |
| A12 | -4 | 52 | 28 | 0 | -48 | 26 | -50 | -60 | 40 | 60 | -52 | 28 |
| A13 | 0 | 56 | 30 | 2 | -54 | 18 | -48 | -66 | 26 | 54 | -56 | 24 |
| A14 | 2 | 48 | 42 | -4 | -50 | 26 | -42 | -66 | 40 | 54 | -56 | 38 |
| A15 | 0 | 48 | 36 | -2 | -50 | 34 | -40 | -62 | 32 | 52 | -64 | 32 |
| A16 | 0 | 46 | 34 | 0 | -50 | 28 | -46 | -62 | 30 | 52 | -60 | 40 |
| A17 | 0 | 54 | 34 | 0 | -54 | 26 | -48 | -64 | 26 | 54 | -64 | 30 |
| A18 | 2 | 50 | 40 | -2 | -54 | 30 | -54 | -60 | 26 | 56 | -62 | 26 |
| A19 | 2 | 44 | 46 | -2 | -52 | 32 | -54 | -62 | 26 | 52 | -62 | 32 |
| A20 | 2 | 54 | 34 | 4 | -60 | 20 | -46 | -70 | 28 | 50 | -66 | 32 |
| A21 | -2 | 46 | 38 | 2 | -60 | 34 | -48 | -64 | 24 | 54 | -60 | 24 |
| A22 | -8 | 40 | 54 | 2 | -54 | 26 | -48 | -64 | 24 | 54 | -60 | 26 |
| A23 | 0 | 48 | 38 | -2 | -50 | 26 | -50 | -68 | 32 | 48 | -68 | 34 |
| A24 | 0 | 38 | 52 | -2 | -56 | 28 | -50 | -66 | 24 | 54 | -60 | 24 |
| A25 | -4 | 48 | 40 | -2 | -58 | 30 | -44 | -68 | 38 | 46 | -70 | 40 |
| A26 | 0 | 52 | 28 | 4 | -54 | 22 | -52 | -52 | 34 | 58 | -58 | 24 |
| A27 | 4 | 40 | 50 | 2 | -54 | 20 | -52 | -62 | 32 | 56 | -62 | 28 |

C = control subjects; B = patients before treatment; A = patients after treatment;

We evaluated the Euclidean distances between ROIs and the center of ROIs. Not all the Euclidean distances yield to the normal distribution. Therefore, we performed Kruskal-Wallis test to the Euclidean distances.

$$c_{i}=\sqrt{\left( x_{i}-\bar{x} \right)^{2}+\left( y_{i}-\bar{y} \right)^{2}+\left( z_{i}-\bar{z} \right)^{2}}$$

Table 2 Mean and standard error of the Euclidean distance

|  | Control | Pre-treatment | Post-treatment | p |
| --- | --- | --- | --- | --- |
| MFC | 8.31±1.210 | 7.38±0.907 | 8.56±1.160 | 0.8602 |
| PCC | 7.69±0.794 | 5.22±0.505 | 6.52±0.554 | 0.0721 |
| LPC | 7.48±0.516 | 7.90±0.761 | 6.77±0.557 | 0.4875 |
| RPC | 8.08±0.692 | 6.85±0.766 | 7.30±0.655 | 0.2694 |


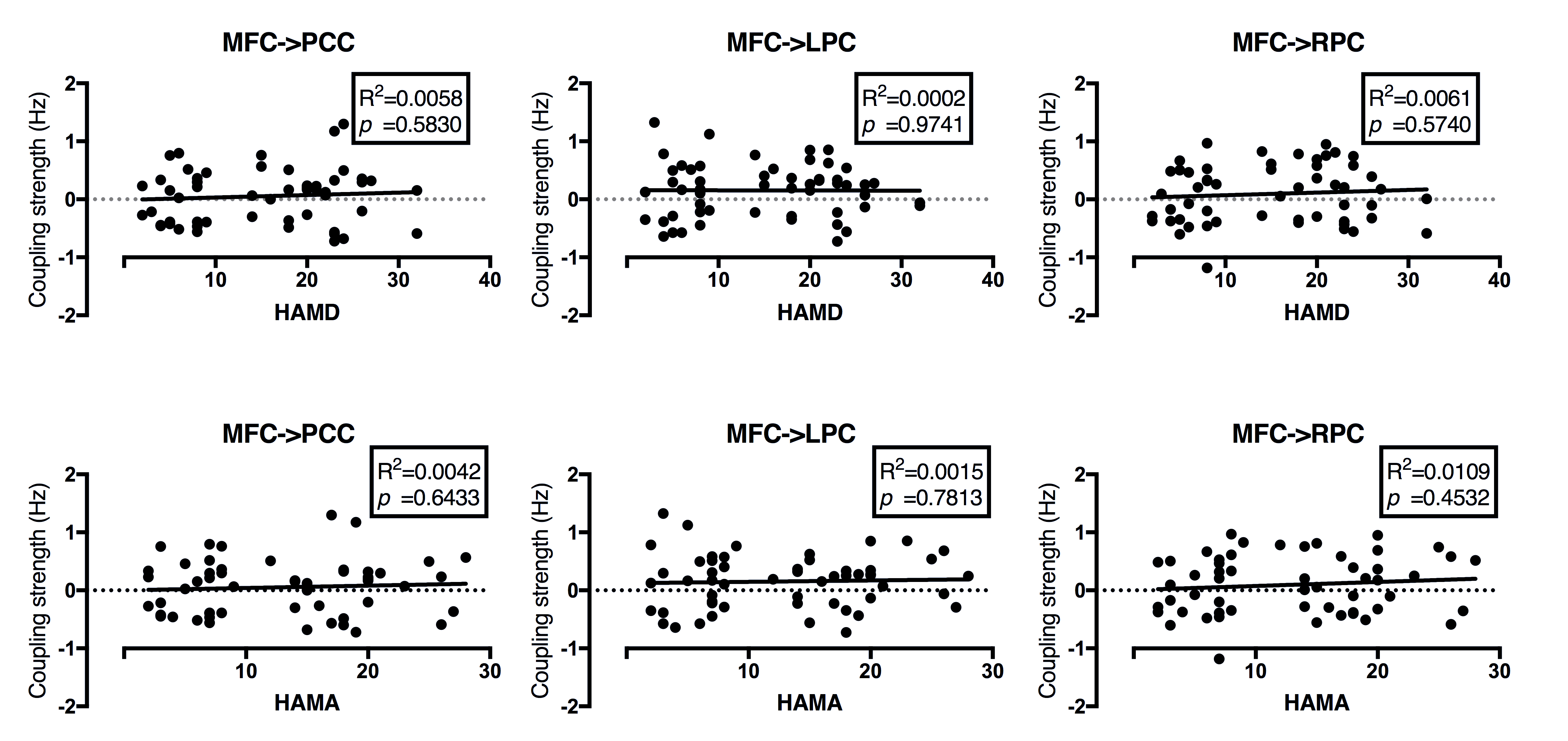


Figure 1. The scatter plots of the coupling parameters(from MFC to other DMN nodes) against the HAMD/HAMA scores in the patients with MDD. The coupling parameters and the corresponding clinical scores of the patients before and after treatment were included.


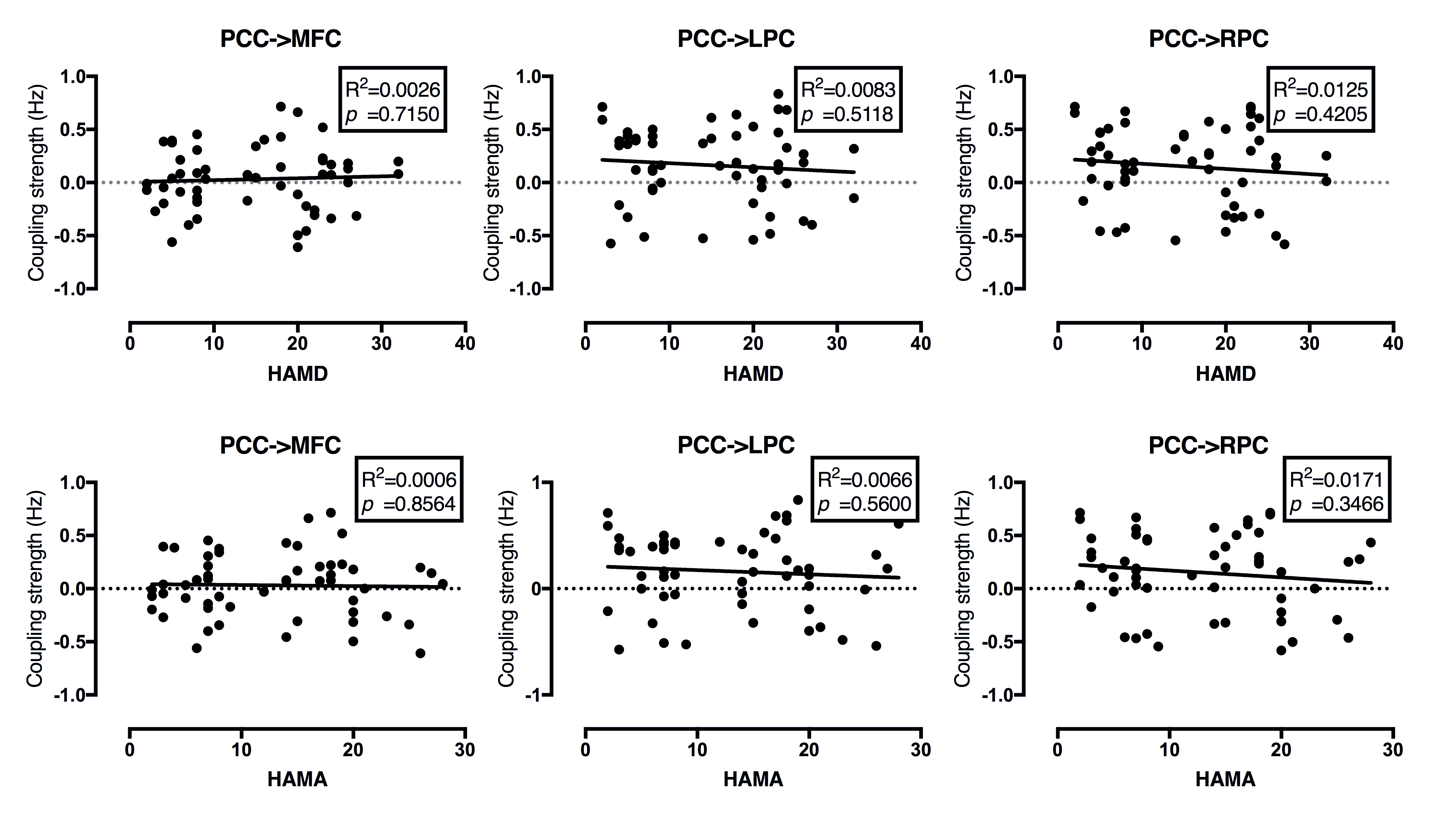


Figure 2. The scatter plots of the coupling parameters(from PCC to other DMN nodes) against the HAMD/HAMA scores in the patients with MDD. The coupling parameters and the corresponding clinical scores of the patients before and after treatment were included.


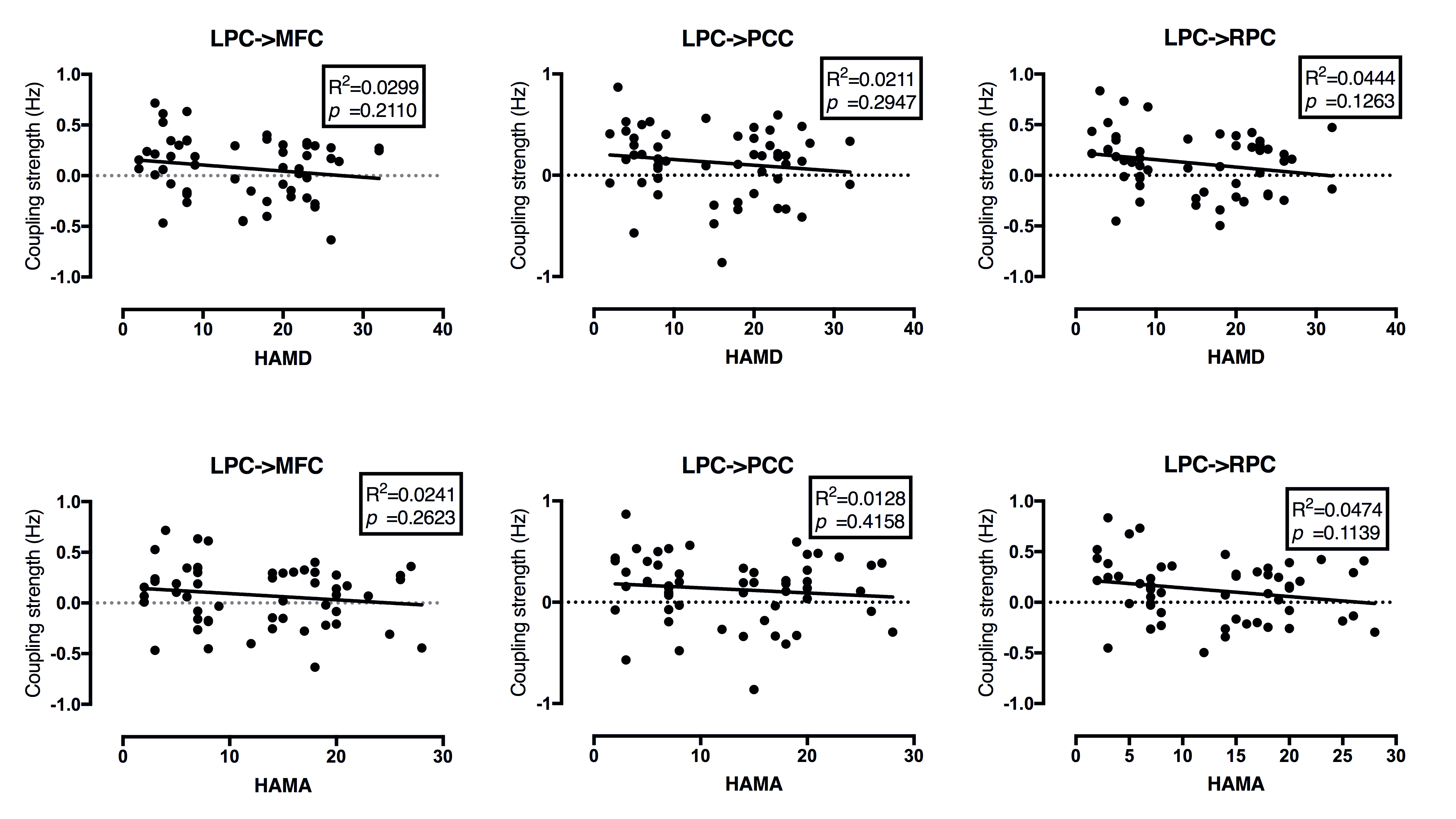


Figure 3. The scatter plots of the coupling parameters(from LPC to other DMN nodes) against the HAMD/HAMA scores in the patients with MDD. The coupling parameters and the corresponding clinical scores of the patients before and after treatment were included.


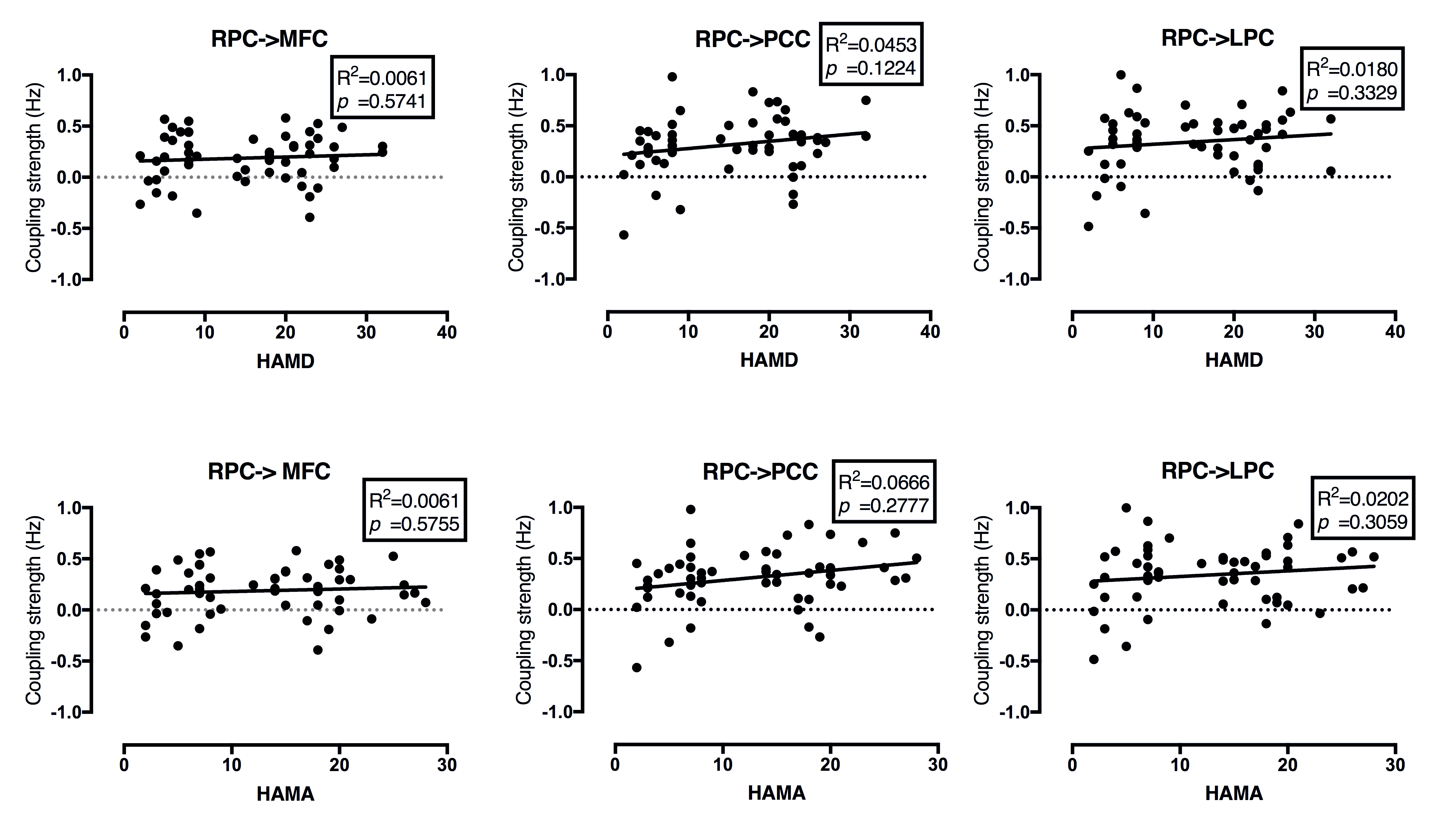


Figure 4. The scatter plots of the coupling parameters(from RPC to other DMN nodes) against the HAMD/HAMA scores in the patients with MDD. The coupling parameters and the corresponding clinical scores of the patients before and after treatment were included.
